# Exosomes From Human Umbilical Cord Mesenchymal Stem Cells Alleviate Oxidative Stress‐Induced POI by Regulating Autophagic Homeostasis Through the AMPK Pathway

**DOI:** 10.1002/rmb2.12658

**Published:** 2025-05-26

**Authors:** Wanqi Chen, Qi Feng, Chan Zhang, Lu Yang, Jingyi Qi

**Affiliations:** ^1^ Luoyang Central Hospital Affiliated to Zhengzhou University luoyang China; ^2^ The Second Clinical Medical School of Zhengzhou University Zhengzhou China

**Keywords:** AMPK, autophagy, EXOs, hu‐MSCs, oxidative stress, POI

## Abstract

**Background:**

Increased oxidative stress is a key factor in developing premature ovarian insufficiency (POI). Exosome therapy emerges as a promising cell‐free treatment. However, research into the molecular mechanisms of exosome repair in ovarian diseases is still in its infancy. By establishing models of oxidative stress in ovarian granulosa cells and POI in mice, we aim to explore whether human umbilical cord mesenchymal stem cell exosomes can repair oxidative damage in ovarian granulosa cells and mouse ovaries, as well as identify potential targets of action. Our goal is to provide new ideas and methods for the clinical application of exosomes and the early prevention and treatment of POI.

**Methods:**

Hydrogen peroxide (H_2_O_2_) (200 μM, 2 h) and D‐galactose (D‐gal) (200 mg/kg, 56 days) were used to induce oxidative stress in ovarian granulosa cells and mice, respectively. Subsequently, exosomes were added to the injury model to validate the mechanism of exosome repair of oxidative damage. We evaluated senescence indicators, AMPK activation, and autophagy.

**Result:**

Through the execution of in vivo and in vitro experiments, it was observed that the activation of the AMP‐activated protein kinase (AMPK) pathway is induced by exosome intervention, leading to a reduction in the accumulation of autophagic vesicles and the restoration of the patency of autophagic flow. This, in turn, results in the repair of oxidative stress‐induced damage and the enhancement of the function of damaged cells and ovaries.

**Conclusion:**

Our findings indicate that exosomes derived from human umbilical cord stem cells have the beneficial effect of ameliorating oxidative stress‐induced POI by activating AMPK and regulating autophagic homeostasis.

## Graphical abstract

We propose for the first time that exosomes from human umbilical cord mesenchymal stem cells (HuMSCs‐EXOs) restore autophagic homeostasis through the AMPK pathway to repair oxidative stress‐induced premature ovarian insufficiency, which is a potential therapeutic for restoring ovarian function in patients with POI.

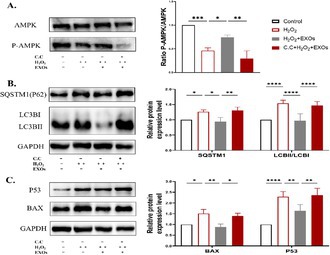

## 1 Introduction

Premature ovarian insufficiency (POI) can be defined as the cessation of ovarian function due to either follicular depletion or the loss of quality of the remaining follicles before the age of 40, and is characterized by infertility, menstrual disorders, and low estrogen levels [1]. Prevalence of the disease in the population is 1%–3% [2]. Estrogen deficiency can lead to a number of symptoms, including hot flashes, insomnia, and memory loss. Additionally, it greatly increases the risk of developing complications such as osteoporosis, cognitive dysfunction, and cardiovascular disease. These complications have been demonstrated to have a considerable impact on women's physical and mental health, and consequently their quality of life.

The etiology of POI is multifaceted and encompasses a range of factors, including genetic predispositions, autoimmune disorders, infectious agents, the consequences of medical interventions, and environmental exposures. Nevertheless, over 50% of POI cases remain idiopathic, lacking an identified etiology [3]. It has been shown that oxidative stress is closely linked to developing POI [4]. Reactive oxygen species (ROS) are a group of molecules produced by the incomplete reduction of oxygen [5], including superoxide radicals, hydroxyl radicals, and hydrogen peroxide, among others. An elevated generation of ROS disrupts the architecture and operational capabilities of nucleic acids, fatty acids, and polypeptides, and induces cellular oxidative stress [6], which leads to aberrant apoptosis of granulosa cells and follicular atresia, affecting female fertility [7].

The clinical treatment strategies for POI encompass lifestyle modification, hormone replacement therapy (HRT), and puberty induction [8]. The objective of these treatments is to enhance the quality of life of patients. However, they are not effective in restoring ovarian function. Despite the utilization of assisted reproductive technologies, it remains impossible to modify pregnancy and reproductive outcomes. Furthermore, HRT has been demonstrated to increase the risk of endometrial, ovarian, and breast cancers [9]. Consequently, there is a necessity to develop new treatment strategies. In recent years, human umbilical cord mesenchymal stem cells (Hu‐MSCs) have gained significant traction in the field of regenerative medicine due to their ease of access and low immunogenicity, which renders them a promising candidate for clinical applications. Recent studies have demonstrated that their derived exosomes are effective in minimizing unfavorable side effects in comparison to stem cells themselves, and are emerging as a promising tool for cell‐free therapy [10, 11]. Human umbilical cord mesenchymal stem cells exosomes (HuMSCs‐EXOs) have demonstrated durable stability in vivo [12]. This advantage not only enhances their potential application but also avoids the problems of low survival rate, immune rejection, and tumorigenic risk that may be associated with direct mesenchymal stem cells transplantation [13]. Exosomes are known to contain a variety of components, including proteins, DNA, mRNA, microRNA, long non‐coding RNAs, and genetic information from viruses or prions, which can be delivered to recipient cells to regulate protein synthesis and signaling pathways [14]. To date, clinical studies have demonstrated the effectiveness of exosome therapy in a range of conditions, including acute respiratory distress syndrome, kidney disease, graft‐versus‐host disease, osteoarthritis, stroke, Alzheimer's disease, and Type 1 diabetes [15]. However, research into the use of exosome therapy in the treatment of ovarian‐related diseases is still in its infancy, and the specific molecular mechanisms by which they act are not yet fully elucidated.

Autophagy is the process by which cells degrade and recycle proteins and organelles in order to maintain intracellular homeostasis. Under usual circumstances, autophagy plays a crucial role in enabling follicular cells to facilitate oocyte maturation, follicular expansion and specialization, follicular regression, and the reproductive lifecycle [16]. However, impaired autophagy in ovarian follicular cells can result in poor egg quality and, consequently, premature ovarian insufficiency in women [17]. AMP‐activated protein kinase (AMPK) is a pivotal regulator of metabolism and energy conversion. It has been demonstrated that ROS are involved in the regulation of the AMPK pathway [5], and that the AMPK signaling pathway modulates autophagy in cells [18].

Therefore, we propose the following hypothesis that HuMSCs‐EXOs restore autophagic homeostasis through the AMPK pathway to repair oxidative stress‐induced premature ovarian insufficiency, which is a promising treatment option for rejuvenating ovarian function in individuals suffering from POI. In our research, we explored the impact of exosomes on POI through two main approaches: (1) examining the shielding effects and underlying mechanisms of HuMSCs‐EXOs on ovarian granulosa cells exposed to hydrogen peroxide (H_2_O_2_), and (2) validating the consequences of exosomes on oxidative stress‐induced damage to mouse ovaries triggered by D‐gal.

## 2 Materials and Methods

### 2.1 Cell Culture

The human ovarian granulosa cell line KGN cells are derived from female ovarian granulosa cell tumors. The human primary umbilical cord mesenchymal stem cells originate from human umbilical cord tissue. Both types of cells were purchased from Shanghai Kuisai Biotechnology Co. Ltd. The human ovarian granulosa cell line KGN cells grow adherently and are cultured in DMEM/F12 complete medium (supplemented with 10% fetal bovine serum and 1% penicillin–streptomycin in DMEM/F12 medium). The human umbilical cord mesenchymal stem cells also grow adherently and are maintained in exosome‐depleted complete medium for human umbilical cord blood mesenchymal stem cells (Cyagen Biosciences Inc., Guangzhou, China). Both cell types are incubated in a 37°C incubator with 5% CO_2_. Routine subculture and cryopreservation procedures are followed.

### 2.2 Collection, Extraction and Characterization of HuMSCs‐EXOs

The method for obtaining exosomes was properly acquired according to minimal information for studies of extracellular vesicles (MISEV2023) guidelines [19]. Well‐grown 3–5 generations of Hu‐MSCs (Shanghai QuiCell Biology, Shanghai, China) were cultured in exosome‐free complete medium (Cyagen Biosciences Inc., Guangzhou, China). The centrifuge tube was utilized to collect the supernatant, which was then subjected to a series of centrifugation steps. Initially, centrifugation was performed at 300 g for 10 min at 4°C, followed by another centrifugation at 2000 *g* for an additional 10 min, both times collecting the supernatant. Subsequently, the supernatant was centrifuged at 10,000 *g* for 30 min at 4°C, once again retaining the supernatant. Finally, a high‐speed centrifugation at 100,000 *g* for 70 min at 4°C was conducted to obtain the precipitate. This precipitate was resuspended in PBS, resulting in the exosome solution. Following aseptic treatment, the exosome solution was stored in a refrigerator at −80°C. The morphology and dimensions of the exosomes were visualized using transmission electron microscopy, while their particle size distribution was analyzed through laser scattering techniques. Furthermore, the presence of surface proteins CD9, CD63, and TSG101 was confirmed via Western blot.

### 2.3 Cell Viability Assay

The ovarian granulosa cells were uniformly distributed in 96‐well plates at a density of 1 × 10^4^ cells per well. After a span of 24 h, the cells were subjected to diverse concentrations of hydrogen peroxide (0–400 μM) for a period of 2 h. Subsequently, 10 μM of CCK‐8 reagent (Suzhou Yuheng, Jiangsu, China) was introduced into each well. After a period of 3 h in the incubator for incubation, the absorbance reading at 450 nm was obtained through the use of an enzyme‐based assay, allowing for the computation of cellular viability. To assess the reparative impact of HuMSCs‐EXOs, cells were incubated with varying concentrations of HuMSCs‐EXOs (0–20 μg/mL) for 24 h following the induction of damage with 200 μM H_2_O_2_ for 2 h. Following the addition of the CCK‐8 solution, the absorbance was measured and the survival rate was calculated.

### 2.4 DCFH‐DA Detection of Intracellular ROS

To ascertain the impact of HuMSCs‐EXOs on oxidatively damaged cells, a DCFH‐DA fluorescent probe was employed. The operation was performed according to the ROS kit (Beyotime, Jiangsu, China). The cells were inoculated in a 6‐well plate at a density of 5 × 10^5^ cells per well and stimulated by the addition of an appropriate concentration of the drug. The cell culture was removed after reaching a predetermined time point. 1 mL of the prepared working solution was aliquoted into each well, and the cells were subsequently incubated in a 37°C cell culture incubator for a duration of 30 min. Subsequently, the cells underwent three washes with serum‐free culture medium. The fluorescent signals were then observed and captured using a 50× magnification fluorescence microscope, with three positions randomly selected in each group to obtain images.

### 2.5 Detection of Cellular Mitochondrial Membrane Potential by JC‐1 Staining

We measured mitochondrial membrane potential by JC‐1 staining. The operation was performed according to the JC‐1 kit (Beyotime, Jiangsu, China). The cells were inoculated in 6‐well plates at a density of 5 × 10^5^ cells per well and stimulated by the addition of appropriate concentrations of drugs. The cell culture was removed after reaching a predetermined time point. A 6‐well plate received a combined total of 1 mL of cell culture solution and 1 mL of JC‐1 staining solution, which were thoroughly mixed. Following a 20‐min incubation at 37°C in a cell incubator, the supernatant was removed by aspiration, and the plates were washed twice using the JC‐1 staining buffer. The samples were then observed under a fluorescence microscope at 200× magnification, with three positions randomly selected in each group to obtain images.

### 2.6 Establishment of Experimental Animal Models

Female C57BL/6 mice, aged 8 weeks and weighing 18–22 g (*n* = 20), were allowed to acclimate for 7 days. Using vaginal smears, mice with regular estrous cycles were chosen and subsequently randomized into three distinct groups (*n* = 5 each). In the normal control group, mice received daily intraperitoneal injections of an equivalent volume of saline. In the POI model, mice underwent daily intraperitoneal administrations of 200 mg/kg of D‐galactose for a period of 56 days. In the exosome group, mice were intraperitoneally injected with 200 mg/kg of D‐galactose daily for 56 days. Starting from the 14th day, they received injections of 150 μg of exosome protein [20] every other day until the end of the experiment. Twenty‐four hours post‐treatment, all mice were euthanized by cervical dislocation. Depending on the experimental requirements, ovaries were harvested and either fixed in 4% paraformaldehyde or stored at −80°C. The present research was granted authorization by the Ethics Committee for Animal Experimentation at Luoyang Central Hospital (No. LWLL‐2024‐11‐13‐02).

### 2.7 Mouse Vaginal Smears

Insert a saline‐moistened swab into the vaginal cavity of the mouse and gently rotate the swab along the vaginal wall. Remove the swab and transfer the cells to the slide by rolling it over the slide. Allow the cells to air dry. Stain each slide with drops of Swiss‐Giemsa stain (Nanjing Jiancheng Biotechnology Research Institute, Nanjing, China). Rinse the floating color with distilled water. Allow the slides to air dry and then observe them under the microscope.

### 2.8 Hematoxylin and Eosin (HE) Staining

Mouse ovary tissues were collected after 24 h of treatment and fixed in 4% paraformaldehyde for 24 h. Following dehydration using gradient alcohol, extraction, wax immersion, and embedding processes, the tissues underwent sectioning. Subsequently, they were baked at 60°C for a duration of 2 h and then deparaffinized utilizing xylene. Dehydration was repeated with a gradient alcohol series, followed by staining with aqueous hematoxylin for a 30‐s interval. Afterward, the sections were stained with eosin for 2 min and examined beneath a light microscope.

### 2.9 Determination of Serum Sex Hormone Levels in Mice

The blood samples were allowed to stand at ambient temperature for 2 h prior to centrifugation at 1000 *g* for a duration of 20 min, followed by the collection of serum in Eppendorf tubes. The concentrations of ovarian function‐related biomarkers in each group were quantitated using specific enzyme‐linked immunosorbent assay (ELISA) kits. The experimental procedure was executed in strict adherence to the manufacturer's instructions for the following kits: the estradiol (E_2_) kit (20231, LEDAQIBO, China), the follicle‐stimulating hormone (FSH) kit (20239), the luteinizing hormone (LH) kit (21468, LEDAQIBO, China), and the anti‐Mullerian hormone (AMH) kit (20514, LEDAQIBO, China).

### 2.10 Assays of Superoxide Dismutase (SOD) and Malondialdehyde (MDA)

The methods of the SOD and MDA kits (Nanjing Jianjian Biotechnology Institute) were followed. The cells of each group and the serum of each group of mice were taken, and the corresponding reagents were added according to the operation table. The different absorbances were read on an enzyme meter, and the corresponding activities were calculated.

### 2.11 Protein Extraction

A solution comprising 1% phenylmethyl sulfonyl fluoride (PMSF), phosphatase inhibitor mixture, and radioimmunoprecipitation (RIPA) lysate was prepared at a ratio of 1:1:50, resulting in a lysate solution. Grouped cells were scraped off from a 6‐well plate, and an appropriate amount of lysate was added. The lysate, steel beads, and ovarian tissue were combined and subjected to grinding in a tissue grinder. The tissue and cells were then disrupted via ultrasound. Centrifugation at 12,000 rpm for 30 min was performed to obtain the protein. The concentration of cell and tissue proteins was determined using a bicinchoninic acid (BCA) kit (Biosharp, Beijing, China).

### 2.12 Western Blot

The PVDF membranes underwent treatment with 5% skim milk powder (Beyotime, Jiangsu, China) for a duration of 1 h. Following this, they were incubated with the primary antibody overnight at a temperature of 4°C. Subsequently, at room temperature, the membranes were incubated for an additional hour with a goat anti‐rabbit IgG secondary antibody. The visualization of the proteins was conducted with the use of an ultrasensitive ECL chemiluminescent solution (NCM Biotech, Jiangsu, China). The primary antibodies utilized in this study were CD9 antibody (25 Kda, 1:1000, Abcam, England), TSG101 antibody (44 Kda, 1:1000, Abcam, England), CD63 antibody (55 K da, 1:1000, Abcam, England), AMPK antibody (62 Kda, 1:1000, CST, America), p‐AMPK antibody (62 Kda, 1:1000, CST, America), P53 antibody (53 Kda, 1:5000, Proteintech, Hubei, China), SQSTM1 antibody (62 Kda, 1:1000, CST, America), BAX antibody (20 Kda, 1:1000, CST, America), LC3B antibody (18, 15 KDa, 1:1000, Proteintech, Hubei, China), and GAPDH antibody (36 Kda, 1:1000, Beyotime, China).

### 2.13 Statistical Analysis

Each experiment was replicated at least three times. Data were reported as mean ± standard deviation. Group differences were analyzed using one‐way ANOVA, followed by Tukey's test. Statistical analyses were performed on GraphPad 9.0 software. Significance was set at *p* < 0.05.

## 3 Results

### 3.1 Characterization of HuMSCs‐EXOs

**FIGURE 1:**
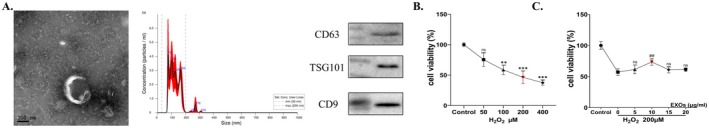
(A) Characterization of HuMSCs‐EXOs (B) Cell viability was assessed using CCK‐8 after H_2_O_2_ treatment at concentrations of 0, 50, 100, 200, and 400 μM. (C) Following a 2‐h incubation period with 200 μM hydrogen peroxide, exosomes (0, 5, 10, 15, 20 μg/mL)were introduced, cell viability was assessed using the CCK‐8 assay. **p < 0.01vs.Control, ##*p* < 0.01 vs. 0 μg/mL EXOs.

A high concentration of exosomes can be successfully isolated by ultracentrifugation, and the formation of white flocculent can be observed at the bottom of the centrifuge tube. The morphology of the exosomes was observed to be teato‐shaped or elliptical double‐membrane structures of varying sizes. The average particle size was determined to be 127.0 ± 0.8 nm, with 96.0% of particles falling within the distribution range of 30–200 nm. Furthermore, Western blot analysis demonstrated that HuMSCs‐EXOs exhibited the expression of specific surface markers, including CD9, CD63, and TSG101 (Figure 1A). These findings align with the characteristics of exosomes as described in the literature.

### 3.2 Establishment of Oxidative Stress Cell Models

The cells were subjected to injury through the application of H_2_O_2_ solutions at concentrations of 0, 50, 100, 200, and 400 μM for a period of 2 h. The viability of the cells was detected by CCK8. The findings revealed a decline in cell viability with an increase in H_2_O_2_ concentration. At a concentration of 200 μM, the cell viability was approximately 50%. Accordingly, the 200 μM concentration was selected as the basis for modeling cell injury (Figure 1B). Subsequently, the cell viability was examined following the administration of exosomes at concentrations of 0, 5, 10, 15, and 20 μg/mL over a 24‐h period. As illustrated in Figure 1C, an increase in exosome concentration resulted in a gradual enhancement in cell viability, reaching a peak and subsequently declining. The highest cell viability was observed at an exosome concentration of 10 μg/mL. For subsequent experiments, a concentration of 10 μg/mL of exosomes was selected as optimal.

### 3.3 HuMSCs‐EXOs Repair Oxidative Stress Damage of Human Ovarian Granulosa Cells

**FIGURE 2:**
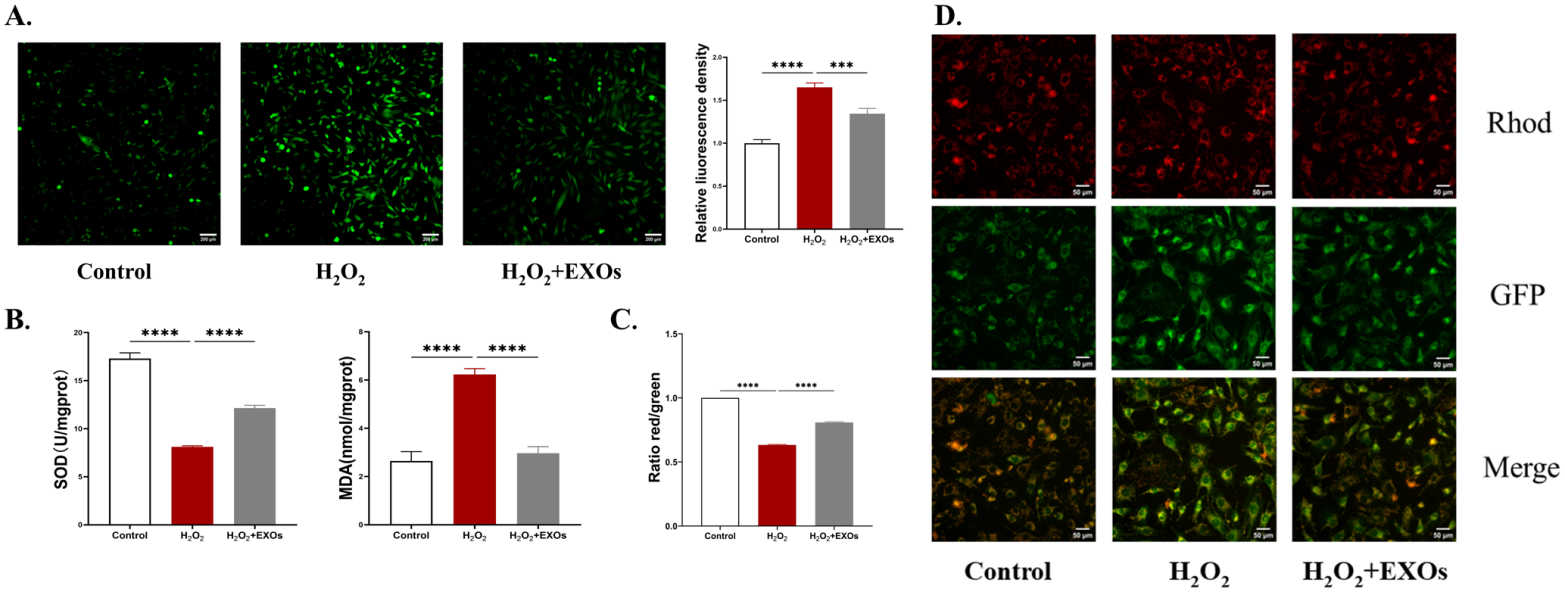
HuMSCs‐EXOs repair oxidative stress damage of human ovarian granulosa cells (A) Observation of intracellular ROS levels by fluorescence microscopy and quantitative analysis of ROS fluorescence density between groups (B) Cellular SOD activity was quantitatively assessed, and MDA levels were determined in each group (C) Intergroup comparison of the red‐to‐green fluorescence intensity ratio was conducted using statistical analysis (D) Fluorescence microscopic observation of fluorescence levels between groups.***p < 0.001, ****p < 0.0001.

We detected intracellular reactive oxygen species levels by fluorescent probes, and the results showed that ROS were significantly increased in the cells of the H_2_O_2_ group compared with the cells of the normal group, and then intracellular ROS were reduced by the addition of exosomes (Figure 2A).

SOD and MDA are important intracellular oxidoreductases. The test results showed that SOD activity was significantly decreased and MDA level was significantly increased in the H_2_O_2_ model group, which could be significantly reversed after repair with exosomes, with increased SOD activity and significantly decreased MDA level (Figure 2B).

In addition, we also measured changes in mitochondrial membrane potential to assess mitochondrial oxidative stress. When mitochondria are damaged by oxidative stress, their respiratory chain electron transfer process may be impaired, which affects the formation of a transmembrane proton (H+) gradient within the matrix, leading to a decrease in mitochondrial membrane potential. Our experimental results revealed a notable decrease in mitochondrial membrane potential in the H_2_O_2_‐induced damage group, marked by intensified green fluorescence and diminished red fluorescence. Conversely, the group treated with exosomes exhibited a considerable rise in mitochondrial membrane potential, characterized by augmented red fluorescence and decreased green fluorescence when compared to the damage group. Analysis of the red‐to‐green fluorescence ratio further confirmed that HuMSCs‐EXOs were capable of elevating the mitochondrial membrane potential in granulosa cells (Figure 2C,D). The above experiments indicate that exosomes can reduce the accumulation of ROS, increase the level of mitochondrial membrane potential, improve the oxidoreductase system, and thus repair the cellular oxidative damage.

### 3.4 Effects of HuMSCs‐EXOs on the Expression of Proteins Related to Oxidative Damaged Cells

**FIGURE 3:**
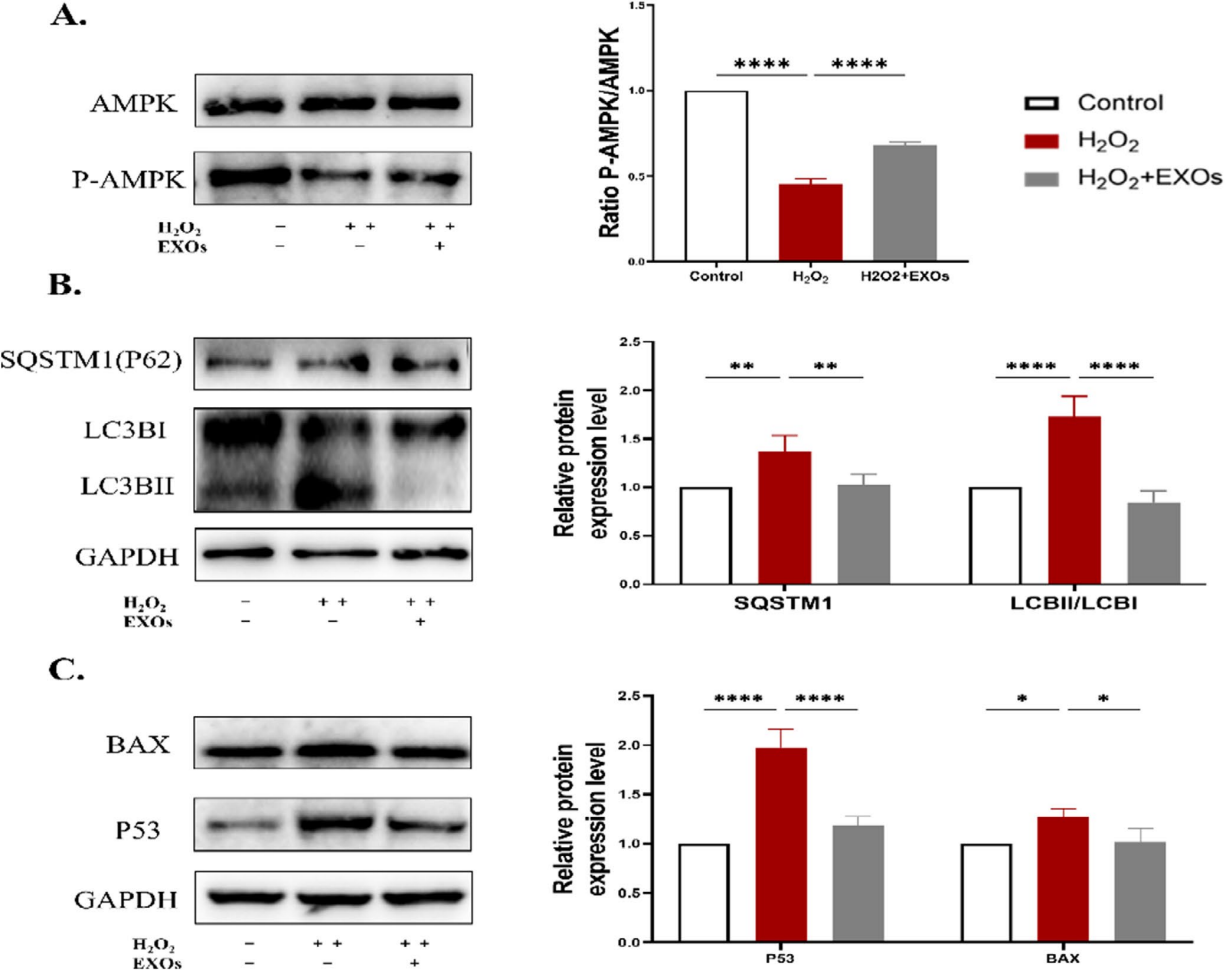
Effects of HuMSCs‐EXOs on the expression of proteins related to oxidative damaged cells (A) Western blot detection of AMPK and P‐AMPK protein expression (B) Western blot analysis was performed to assess the protein levels of autophagy markers LC3B and SQSTM1 (C) Western blot detection of BAX and P53 protein expression. *p < 0.05, **p < 0.01, ***p < 0.001, ****p < 0.0001.

To evaluate the effect of HuMSCs‐EXOs on oxidatively damaged cells at the molecular level, we analyzed the protein expression of AMPK, phosphorylated AMPK (P‐AMPK), autophagy markers LC3B and SQSTM1, and apoptosis‐related factors P53 and BAX using western blot assays. Our findings revealed a notable reduction in P‐AMPK expression in hydrogen peroxide‐injured granulosa cells, in contrast to normal cells. Conversely, the introduction of exosomes led to an elevation in P‐AMPK protein levels in the repaired granulosa cells (Figure 3A). Compared with the control group, the ratio of LC3B‐II to LC3B‐I and the expression of SQSTM1 protein in the model group showed a significant elevation, indicating the accumulation of autophagic microsomes and poor autophagic flow. After treatment with exosomes, the expression of LC3BII/I and SQSTM1 was significantly decreased, and the autophagic flux was restored (Figure 3B). Meanwhile, the protein expression levels of BAX and P53 were significantly higher in the cells after hydrogen peroxide injury, while the expression of BAX and P53 protein levels was reduced in the cells with the addition of exosomes (Figure 3C). Considering the experimental outcomes, we postulated that exosomes decrease H_2_O_2_‐mediated apoptosis through modulation of autophagic balance, potentially linked to AMPK activation.

### 3.5 Effect of the AMPK Inhibitor Compound C (C.C) on Exosome Repair of Oxidative Damage in Granulosa Cells

To investigate the role of the AMPK pathway during oxidative stress in exosome‐repaired granulosa cells, we pretreated the cells with the AMPK inhibitor C.C. In subsequent experiments, the cells were divided into four groups: (1) Normal control group: Cells that did not undergo any additional treatment. (2) H_2_O_2_ induced model group: The cells were treated with 200 μM H_2_O_2_ for a period of 2 h. (3) H_2_O_2_ + EXOs group: Cells were treated with 200 μM H_2_O_2_ for 2 h and then 10 μg/mL HuMSCs‐EXOs for 24 h. (4) C.C + H_2_O_2_ + EXOs group: The cells were initially treated with 50 μM C.C for 2 h, followed by 200 μM H_2_O_2_ for 2 h, and subsequently with 10 μg/mL HuMSCs‐EXOs for 24 h.

#### 3.5.1 The Effect of C.C on Intracellular ROS Levels

**FIGURE 4:**
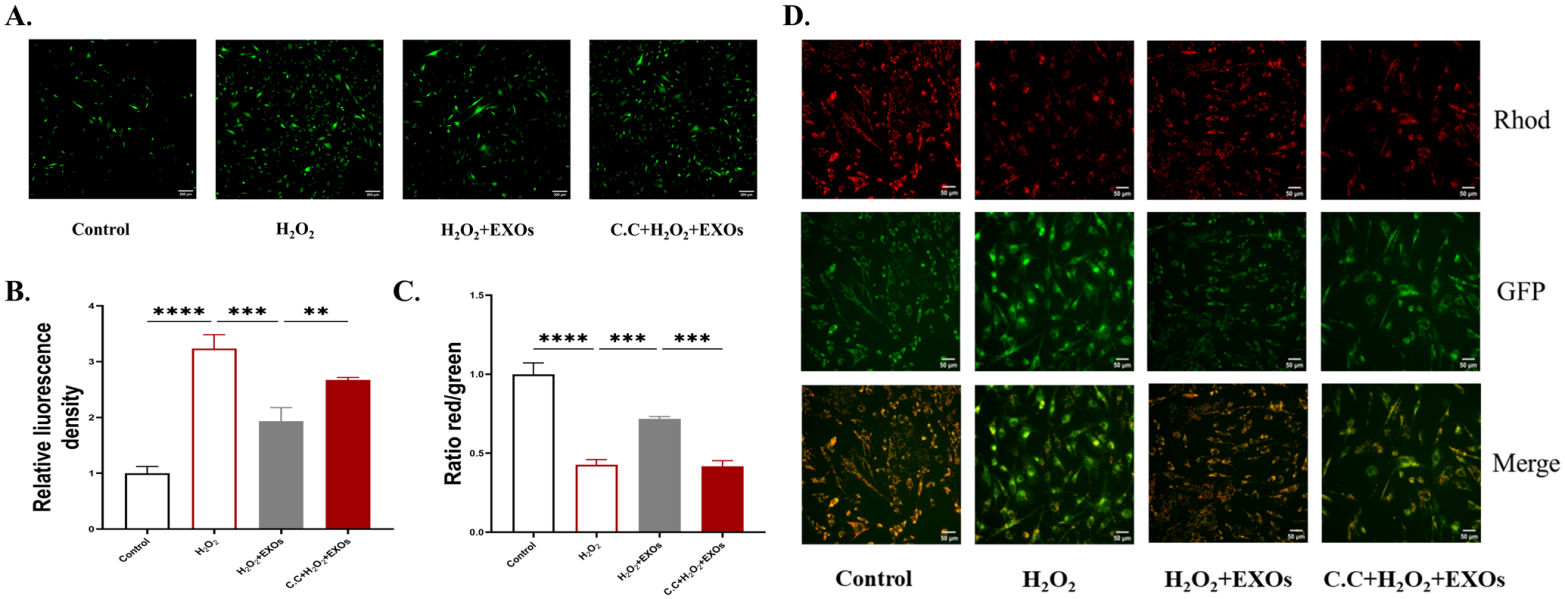
C.C reversed the reduction of intracellular ROS levels and elevation of cellular mitochondrial membrane potential induced by HuMSCs‐EXOs (A) Observe the intracellular ROS levels between groups under the microscope (B) Quantitatively analyze the ROS fluorescence intensity between groups. (C) Quantitative analysis of the ratio of red and green fluorescence intensities between each group (D) Confocal microscope observation of cellular mitochondrial membrane potential between different treatment groups. *p < 0.05, **p < 0.01, ***p < 0.001, ****p < 0.0001.

The intracellular ROS levels were quantified using the DCFH‐DA fluorescent probe. The results demonstrated that the intracellular ROS levels were markedly elevated in the model group relative to the normal group. The administration of HuMSCs‐EXOs led to a notable reduction in ROS levels. However, when the AMPK inhibitor C.C was introduced, the intracellular ROS levels exhibited a significant elevation. These findings suggest that the pretreatment with C.C effectively reversed the reduction in ROS levels by HuMSCs‐EXOs (Figure 4A,B).

#### 3.5.2 Effect of C.C On Cellular Mitochondrial Membrane Potential

We used JC‐1 staining to detect the level of cellular mitochondrial membrane potential between groups. The results showed that the intracellular mitochondrial membrane potential level decreased after injury induction with H_2_O_2_, increased after treatment with HuMSCs‐EXOs, and decreased again after inhibition of AMPK with C.C. Treatment with C.C reversed the protective effect of HuMSCs‐EXOs on mitochondrial membrane potential (Figure 4C,D).

#### 3.5.3 The Impact of C.C on Autophagy and Apoptosis Proteins

**FIGURE 5:**
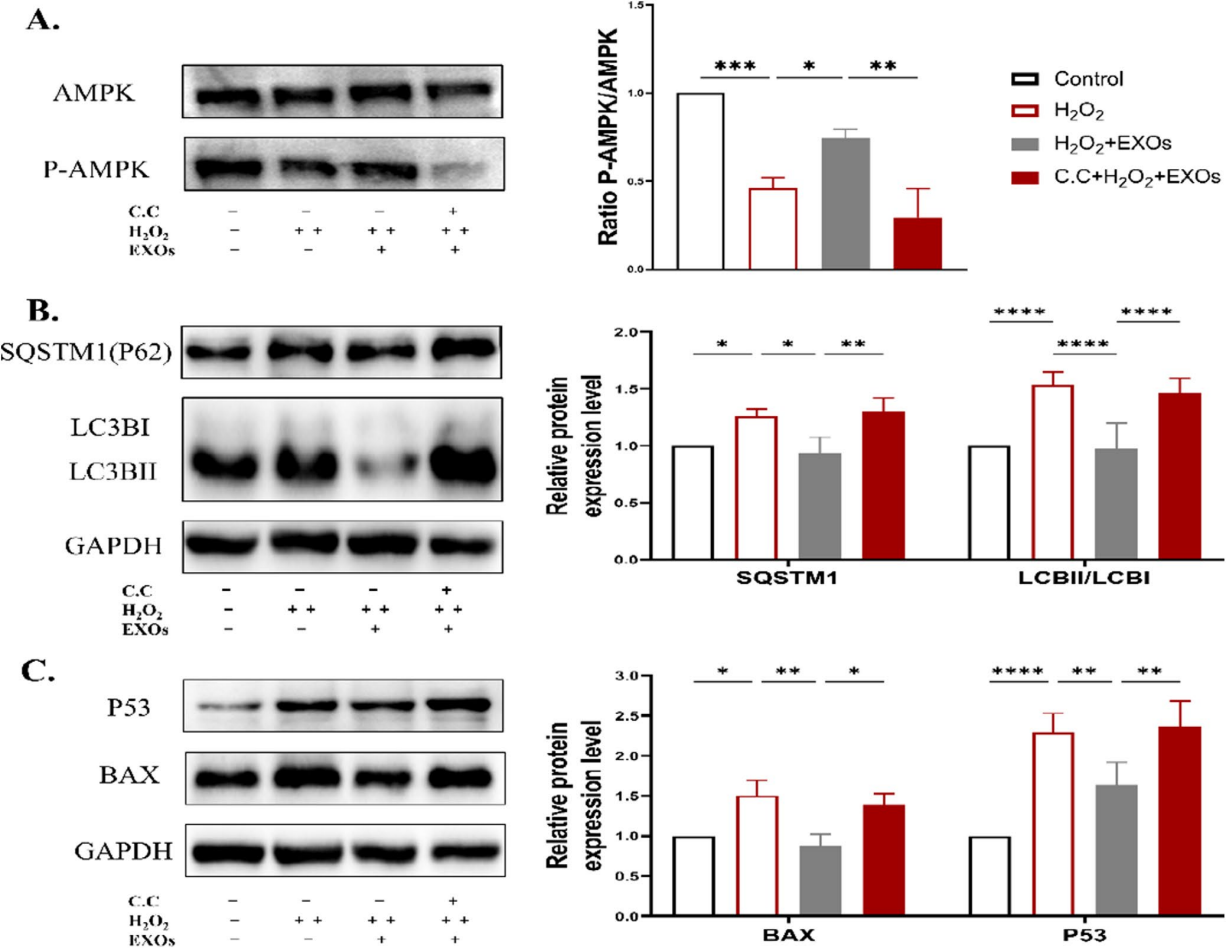
Effects of C.C on the related proteins of AMPK、autophagy and apoptosis (A) Western blot detection of AMPK and P‐AMPK protein expression (B) Western blot analysis was conducted to measure the expression levels of autophagy‐associated proteins, namely LC3B and SQSTM1 (C) Western blot detection of BAX and P53 protein expression. *p < 0.05, **p < 0.01, ***p < 0.001, ****p < 0.0001.

The related proteins were detected by Western blot, and the results demonstrated that C.C markedly inhibited the phosphorylation of AMPK (Figure 5A) and reversed the reparative effect of exosomes on injured granulosa cells, as evidenced by the reincrease of autophagy proteins SQSTM1 and LC3BII/LC3BI (Figure 5B) and apoptotic proteins P53 and BAX (Figure 5C). These findings suggest that HuMSCs‐EXOs activate the AMPK pathway, thereby restoring autophagy homeostasis and alleviating oxidative damage in human ovarian granulosa cells.

### 3.6 Effects of HuMSCs‐EXOs on Body Weight and Estrous Cycle in Mice

**FIGURE 6:**
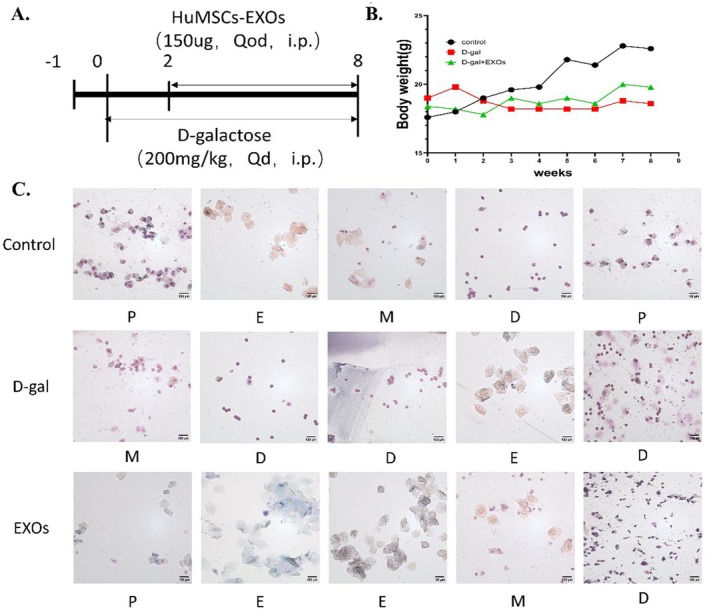
Effects of HuMSCs‐EXOs on body weight and estrous cycle in mice (A) Diagrammatic illustration of the experimental design (B) Weights of the mice recorded (C) Estrous cycles of each group of mice.

Conduct animal experiments according to the schematic diagram of the experimental plan (Figure 6A). The weight of the animals was measured weekly until the end of the experiment. We can see that the mice in the control group had the fastest weight growth, while the mice in the galactose group had an overall decreasing trend in body weight, and the mice in the exosome group had a slow growth in body weight (Figure 6B). The estrous cycle in mice is typically 4–5 days and occurs cyclically in the order of proestrus, estrus, metestrus, and diestrus. Irregularity of the estrous cycle, including disruption, prolongation, or stagnation, is one of the assessment indicators of ovarian insufficiency. The results of the study showed that the mice in the POI model group had estrous cycle disorder, which manifested as prolonged, stalled, or no obvious cycle, and the cycle was improved after treatment with exosomes (Figure 6C).

### 3.7 HuMSCs‐EXOs Restore Ovarian Function in POI Mice

**FIGURE 7:**
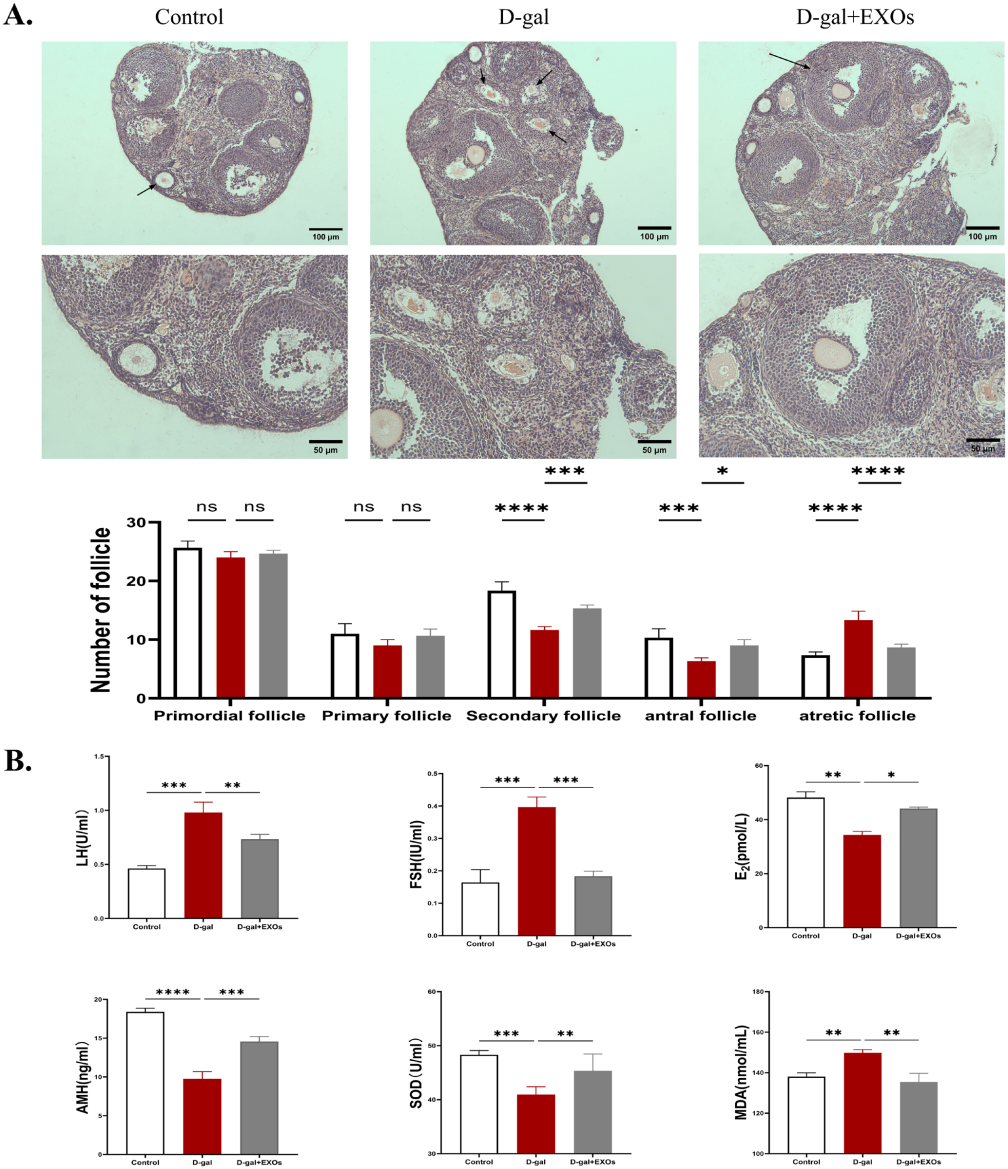
HuMSCs‐EXOs restore ovarian function in POI mice (A) Histological alterations of ovary were observed by HE staining (100X, 200X) (B) ELISA was employed to assess the serum concentrations of ovarian function‐associated factors (E2, FSH, LH, AMH) as well as oxidative stress markers (MDA and SOD) *p < 0.05, **p < 0.01, ***p < 0.001, ****p < 0.0001.

To further investigate the therapeutic effect of HuMSCs‐EXOs on POI mice, we performed histological examination of HE staining of ovaries in each group and counted follicles at all levels. We observed a significant decrease in the count of secondary and sinus follicles, accompanied by an increase in atretic follicles in the model group relative to the control group. After exosome treatment, the ovarian histomorphology was improved compared with the model group, and the number of secondary follicles and sinus follicles increased to some extent, and the number of atretic follicles decreased (Figure 7A).

Furthermore, we analyzed the serum sex hormone concentrations and oxidative stress markers in all groups. Notably, the FSH levels in the POI group declined to nearly match those of the control group following treatment with HuMSCs‐EXOs, while E_2_, LH, and AMH levels exhibited marked improvement. Regarding serum SOD levels, a notable decrement was observed in the model group when compared to the normal control. Conversely, the exosome‐treated group demonstrated a significant elevation in SOD levels compared to the model group. In terms of serum MDA levels, the model group exhibited a significant increase relative to the normal control. However, HuMSCs‐EXO treatment led to a substantial reduction in MDA levels within the ovarian tissues (Figure 7B). Taken together, our data suggest that HuMSCs‐EXOs can improve the oxidoreductase system, repair oxidative stress, and restore ovarian function in POI mice.

### 3.8 HuMSCs‐EXOs Repair Oxidative Damage in Mice via AMPK Pathway

**FIGURE 8:**
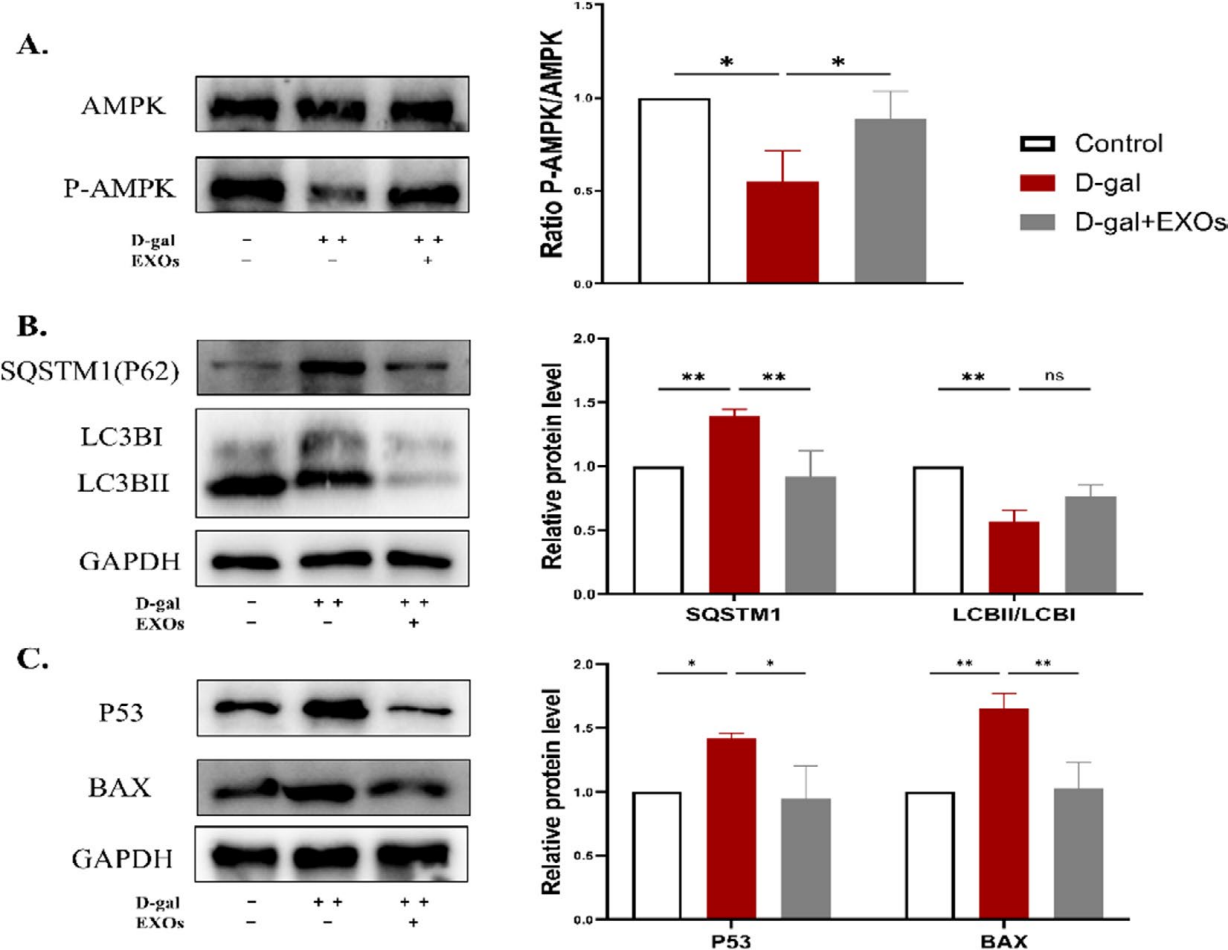
Effect of exosomes from human umbilical cord mesenchymal stem cells on the expression of proteins associated with oxidative damage to the ovaries (A) Western blot detection of AMPK and P‐AMPK protein expression (B) Western blot to detect the protein expression levels of autophagy‐related proteins LC3B and SQSTM1 (C) Western blot detection of BAX and P53 protein expression. *p < 0.05, **p < 0.01, ***p < 0.001.

To verify the mechanism of exosomes repairing oxidative damage in the ovary, Using western blot analysis, we examined the expression levels of AMPK and P‐AMPK, autophagy‐associated proteins LC3B and SQSTM1, as well as apoptosis‐related proteins P53 and Bax in the ovaries of mice. Our findings revealed that the POI model exhibited a marked reduction in the phosphorylation of AMPK, whereas the exosome‐treated group demonstrated a significant elevation in ovarian P‐AMPK protein expression (Figure 8A). Compared with the control group, LC3BII/I protein expression was decreased, SQSTM1 protein expression level was increased, and autophagic flux was not smooth in the model group, and after treatment with exosomes, LC3BII/I expression was increased, SQSTM1 expression was significantly decreased, and exosomes restored autophagic flux to smooth (Figure 8B). In addition, the levels of BAX and P53 proteins were significantly elevated in ovaries with galactose‐induced injury. In contrast, the introduction of exosomes led to a decrease in the expression of BAX and P53 proteins (Figure 8C). In conclusion, exosomes can restore autophagic homeostasis through the AMPK pathway to repair oxidative stress injury in ovaries.

## 4 Discussion

POI is a significant cause of infertility, characterized by diminished ovarian function [21]. The prevalence of late marriage and childbearing is increasing annually, driven by mounting pressures in life. Concurrently, the fertility aspirations of women in their later years are rising, as they seek to enhance their reproductive function to address their unmet fertility needs. Consequently, researchers in this field have dedicated themselves to exploring and elucidating the pathological mechanisms of POI and investigating innovative therapeutic avenues. However, despite their efforts, there is currently no satisfactory therapeutic strategy to alleviate POI, and the development of a new therapeutic strategy to treat POI is imminent.

It has been demonstrated that oxidative stress can mediate alterations in genetic material, signaling pathways, transcription factors, and the ovarian microenvironment, leading to abnormal apoptosis and meiotic abnormalities in ovarian granulosa cells, as well as a decrease in mitochondrial deoxyribonucleic acid, which accelerates the process of ovarian aging [22]. Consequently, in this study, ovarian granulosa cells were utilized to establish an in vitro oxidative damage model to simulate the pathogenesis of POI caused by abnormal oxidative stress. Hydrogen peroxide solution is a commonly used stimulus for oxidative stress in in vitro experiments, so we used 200 μM hydrogen peroxide to act on granulosa cells and successfully established a cellular oxidative stress model. Exosomes can repair oxidative damage in granulosa cells by decreasing ROS in damaged cells, increasing mitochondrial membrane potential levels, increasing SOD activity, and decreasing MDA accumulation.

A consensus on the methodology for constructing animal models of POI remains elusive. The prevailing approach involves the induction of chemotherapy, though the brevity of the induction period for chemotherapeutic agents hinders their capacity to emulate the protracted onset of human ovaries. D‐galactose is a reducing sugar that produces acetaldehyde and hydrogen peroxide under the action of galactose oxidase, causing excessive ROS accumulation and cellular senescence [23]. Since ROS‐induced oxidative damage leading to ovarian tissue decompensation is very similar to the human aging process. Therefore, this study utilized D‐galactose, administered to mice over a 56‐day period, to model POI caused by accumulation of ROS in humans. After the 14th day of injury, we injected exosomes intraperitoneally into the injured mice every other day to establish an exosome model. The results demonstrated that the administration of exosome therapy resulted in an increase in SOD activity and a decrease in MDA accumulation in the serum of mice afflicted with POI. Concurrently, the serum hormone levels of the mice were restored. Pathological examination revealed a notable decline in the count of secondary and antral follicles, coupled with an increase in atretic follicles within the POI model group. Post‐exosomal treatment, however, observed an increase in the number of secondary and antral follicles, along with a decrease in atretic follicles. These experiments showed that ovarian function was restored after exosome injection in mice.

Autophagy is a cellular self‐renewal process that is responsible for the removal of misfolded or abnormally accumulated proteins, damaged organelles, and potential pathogens from cells. This process plays an indispensable role in the maintenance of cellular homeostasis and the prevention of diseases [24]. However, defective autophagy in ovarian follicular cells can lead to poor egg quality and thus female infertility [25]. Autophagy consists of five steps, namely nucleation, elongation, maturation, fusion, and lysosomal degradation [24]. Abnormalities in any of these steps can lead to abnormal autophagy, resulting in cell damage or even death. Therefore, maintaining a smooth flow of autophagy is essential for the normal functioning of cells. During the elongation and maturation of autophagosomal vesicles, LC3 precursor (pro‐LC3) is converted by autophagy‐associated protein 4 (Atg4) to LC3‐I, which is then conjugated to phosphatidylethanolamine (PE) via the activating enzyme ATG7 and the conjugating enzyme ATG3 to form LC3B‐II [24]. Thus, LC3 conversion reflects the progression of autophagy. The primary function of Sequestosome 1 (SQSTM1 or p62) is to deliver various ubiquitinated substances to the autophagosome, thereby facilitating their subsequent degradation by lysosomes. Notably, SQSTM1 itself is subject to degradation via autophagy [26]. Therefore, SQSTM1 is used together with LC3B as a marker of autophagic flux [27]. In this study, the effect of exosome on autophagy was investigated through the application of western blot analysis. The results demonstrated that SQSTM1 expression levels were significantly elevated in the oxidative damage model, suggesting that oxidative stress injury impeded the progression of autophagy and led to an accumulation of autophagic vesicles, consequently resulting in cellular and ovary damage. Conversely, the exosome group exhibited a decline in SQSTM1 protein expression, suggesting that exosome facilitates the uninterrupted progression of autophagy and effectively mitigates the accumulation of autophagosomes, thereby alleviating the oxidative damage induced by autophagy dysfunction.

The structure of AMPK, a heterotrimeric protein complex, contains an α‐subunit with catalytic activity and two subunits that play a regulatory role, the β and γ subunits. This specific combination of subunits enables different types of AMPK complexes to respond accordingly to various stress stimuli [18]. The AMPK pathway has been identified as a classical signaling pathway that regulates autophagy [28]. Through our study, we found that the damage caused by oxidative stress significantly inhibits the expression of the AMPK pathway, autophagy is blocked, and apoptotic proteins are increased. Conversely, exosome treatment increases the expression of phosphorylated AMPK, restores autophagic flow, and significantly reduces apoptotic proteins. Therefore, we hypothesized that the AMPK pathway could repair oxidative stress injury by improving autophagic flow. To test this hypothesis, we pretreated cells with the AMPK inhibitor C.C. Our results showed that the effects of the treatment with exosomes in reducing ROS accumulation and restoring mitochondrial membrane potential were reversed. Concurrently, the expression of apoptotic and autophagy proteins increased, and the reparative effects of the exosome treatment on autophagy flow and oxidative damage were lost. Therefore, we concluded that the exosome treatment restored the patency of autophagy flow by regulating the AMPK signaling pathway, which in turn promoted the recovery of ovarian function.

In conclusion, the results of this study provide preliminary evidence that HuMSCs‐EXOs play a novel role in repairing oxidatively damaged POI by regulating autophagy and that AMPK plays an important role in the regulation of autophagy by exosomes. The number of patients suffering from POI is increasing with the increase in life stress, the increase in ovarian diseases, and the increase in the number of patients undergoing chemotherapy. It is expected that this study will provide a novel theoretical basis and practical approach for the application of exocytosis in the clinical field and promote the restoration of fertility in patients with POI. To the best of our knowledge, this is the first report that exosomes from human umbilical cord MSCs activate the AMPK pathway to regulate autophagy to reduce oxidative stress‐induced POI, but our experiment still has the following shortcomings: (1) only AMPK‐expressed proteins were examined in the present study, and the exosome components and their downstream pathways affecting the AMPK pathway were not explored in depth; (2) the animal experimental part of the study only performed observational validation. The long‐term effects on the safety of the offspring after treatment were not addressed.

## Ethics Statement

The animal experiments were approved by the Ethics Committee for Animal Experimentation at Luoyang Central Hospital (No. LWLL‐2024‐11‐13‐02), and all animal procedures were performed in accordance with institutional and national guidelines.

## Conflicts of Interest

The authors declare no conflicts of interest.
